# Poly(*N*-vinylimidazole): A biocompatible and biodegradable functional polymer, metal-free, and highly recyclable heterogeneous catalyst for the mechanochemical synthesis of oximes

**DOI:** 10.3906/kim-2107-44

**Published:** 2021-10-07

**Authors:** Hayedeh GORJIAN, Hoda FAHIM, Nader GHAFFARI KHALIGH

**Affiliations:** 1 Department of Food Science and Technology, Sari Agricultural Sciences and Natural Resources University, Sari, Iran; 2 Nanotechnology and Catalysis Research Center, Institute for Advanced Studies (IAS), University of Malaya, Kuala Lumpur, Malaysia

**Keywords:** Heterogeneous catalysis, functional polymer, condensation reaction, ball milling technique, solvent-free conditions

## Abstract

The catalytic activity of poly(*N*-vinylimidazole), a biocompatible and biodegradable synthetic functional polymer, was investigated for the synthesis of oximes as an efficient, halogen-free, and reusable heterogeneous catalyst. The corresponding oximes were afforded in high to excellent yields at room temperature and in short times using the planetary ball mill technique. Some merits, such as the short reaction times and good yields for poorly active carbonyl compounds, and avoiding toxic, expensive, metal-containing catalysts, and hazardous and flammable solvents, can be mentioned for the current catalytic synthesis of the oximes. Furthermore, the heterogeneous organocatalyst could be easily separated after the reaction, and the regenerated catalyst was reused several times with no significant loss of its catalytic activity.

## 1. Introduction

Functional polymers are macromolecules containing functional groups [1]. Poly(*N*-vinylimidazole) (PVIm) is a biocompatible [2], biodegradable [3], thermal stable [4], and water-soluble linear polymer with pK_a_ around 6.0 [5]. PVIm, as a pH-sensitive functional polymer, can be protonated at acidic pH value and deprotonated under basic conditions [6]. PVIm and its copolymers have been employed in different engineering applications [7]. PVIm and its copolymers have been applied in the suppressing gene expression, as drug and protein delivery carriers [5], heavy metal removal *via* metal-binding chelating [8,9], catalysis [10–13], pervaporation [14], fuel cell [15], CO_2_ separation [16], and nanofiltration separation [17].

Oximes constitute not only an efficient method for the protection, isolation, purification, and characterization of carbonyl compounds [18] but also have a vital role in the preparation of dyes, fragrances, pharmaceuticals, fungicides, herbicides, and agricultural chemicals [19]. They are extensively used as intermediates for the synthesis of various nitrogen-containing compounds including nitriles [20], nitrones [21], amides [22], hydroximinoyl chlorides [23], azaheterocycles [24], nylon-[6] [25], and act as versatile ligands for metal complexes [26]. Oxime compounds have been used as antidotes for nerve agents [27].

Oximes are often prepared by stirring the carbonyl compounds with NH_2_OH·HCl in the presence of pyridine in ethanol under reflux conditions [28]. This approach has several disadvantages, including (a) the slow reaction rate and low yield of the desired products, (b) pyridine is a toxic and flammable liquid with a strong odor, and (c) the use of organic solvents cause effluent pollution.

Hydroxylamine as the intermediate plays a crucial in organic chemistry. Due to the hazardous properties of hydroxylamines, such as irritation of the eyes, the skin, mucous membranes, and mutagen and allergy activities, their handling and storage required particular attention and facilities. Furthermore, it can explode during its decomposition in the presence of metal ions such as Fe^2+^ and Fe^3+^ or the high basicity of media [29]. Temperature > 65 ºC is the critical temperature for the explosive decomposition of hydroxylamine [30].

**Scheme 1:**
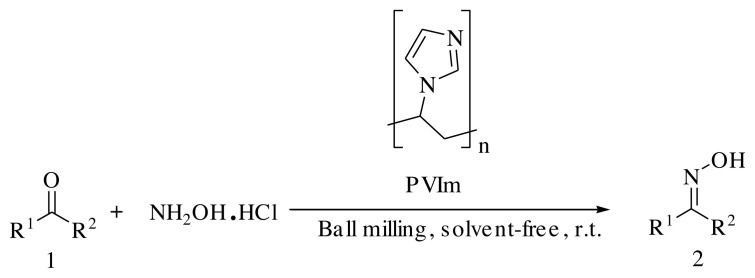
Conditions for the preparation of oximes using PVIm.

To overcome the mentioned disadvantages, the research is going on to find a safe and green methodology using stable and reusable catalysts under mild conditions. Herein, we wish to report a facile mechanochemical condensation of hydroxylamine hydrochloride salt (NH_2_OH·HCl) with R^1^R^2^C=O compounds in the presence of PVIm under solvent-free conditions at room temperature using ball milling technique (Scheme 1).

## 2. Materials and methods

### 2.1. General

The chemicals, reagents, and solvents were of analytical grade and purchased from Sigma Aldrich, ACROS organic, Alfa Aesar, and Fisher Chemical Companies and used as purchased. The purity determination of the products was accomplished by TLC on silica gel polygram SIL G/UV 254 plates. The MS was measured under GC (70 eV) conditions. The IR spectra were recorded on a Perkin Elmer 781 Spectrophotometer. In all cases, the ^1^H NMR spectra were recorded with Bruker Avance 400 MHz instruments. Chemical shifts are reported in parts per million in CDCl_3_ with tetramethylsilane as an internal standard.

### 2.2. General procedure for the synthesis of oximes

PVIm was fabricated through free radical polymerization of *N*-vinylimidazole in toluene at N_2_ atmosphere with azobisisobutyronitrile (AIBN) as the initiator [12]. The M_v_ value of PVIm was determined to be 310,000 g mol through viscometry using the Mark–Houwink–Sakurada equation [31]. The reaction was carried out using two stainless steel balls with a 7 mm diameter at room temperature. The carbonyl compound (5 mmol), NH_2_OH·HCl (6.0 mmol), and poly(4-vinylimidazole) (250 mg) were ground by a planetary ball mill (Retsch PM100) for the appropriate time. After completion of the reaction, toluene (5 mL) was added to the reaction mixture, and the catalyst was separated by simple filtration and washed with toluene (2 × 5 mL) and dried. The solvent was then removed under vacuum by a rotary evaporator, and the pure products were obtained by recrystallization from hot ethanol. The synthesized compounds’ melting point and ^1^H NMR spectra were in good agreement with those reported in the literature [32–36].

### 2.3. Color and ^1^H NMR data of the selected products

*Benzaldehyde oxime (2a)*: pale yellow solid; ^1^H NMR (CDCl_3_, 400 MHz): δ = 9.84 (br s, 1H), 8.14 (s, 1H), 7.68–7.57 (m, 2H), 7.48–7.41 (m, 3H) ppm.

*4-Nitrobenzaldehyde oxime (2d)*: pale orange solid; ^1^H NMR (CDCl_3_, 400 MHz): δ = 9.94 (br s, 1H), 8.14 (s, 1H), 8.35 (d, *J* = 8.8 Hz, 2H), 7.91 (d, *J* = 8.8 Hz, 2H) ppm.

#### 2.3.1. Recovering and reusing of PVIm

PVIm was filtered and washed with hot ethanol (2 × 5 mL) and then dried overnight at 80 ºC by a vacuum oven. The study of FT-IR spectra of fresh and 4th recovered PVIm demonstrated the chemical and thermal stability of PVIm during the reaction, workup, and recycling conditions.

## 3. Results and discussion

Developing cost-effective and eco-friendly processes and performing reactions with safe and greener reagents, solvents, and catalysts are crucial steps of organic synthesis research. In continuation of our recent studies [10–12], herein, the efficient catalytic activity of PVIm, as a functional polymer, for the synthesis of various oximes is described.

Solvent-free condensation of 4-nitrobenzaldehyde and NH_2_OH·HCl was chosen as a model reaction for finding the optimal conditions. Initially, the model reactants were ground together in a mole ratio of 1:1.2 for 4-nitrobenzaldehyde and NH_2_OH·HCl, respectively, at room temperature for one hour by a planetary ball mill. Then, the reaction mixture was left to stand overnight at room temperature (Table 1, entry 1). The starting aldehyde was detected after one hour (monitored by TLC), which approve the failure of the reaction in the absence of a catalyst. Then, the model reactant was milled in the presence of PVIm using a planetary ball mill for 30 min, and the reaction mixture was left to stand about 30 min at room temperature. The GC-MS analysis of the product exhibited a mixture of 4-nitrobenzaldehyde (43%) and 4-nitrobenzaldehyde oxime (67%) (Table 1, entry 2). Then, the amount of catalyst and reaction time were investigated, and, as one can see from the results, both parameters play a vital role in condensing model reactants (Table 1, entries 3–7). The yield increased to 94% when 250 mg of catalyst was used (Table 1, entry 6). The prolonging of the reaction time to one hour showed the same effect (Table 1, entry 7). However, PVIm loading greater than 250 mg shows no improvement in the yield compared with the previous experiment (Table 1, entry 8). The best yield was obtained through the ball milling of the aldehyde/NH_2_OH·HCl in the mole ratio of 1:1.2 in presence of 250 mg PVIm at room temperature within 30 min.

The substrate scope of the new catalytic synthesis of oximes was investigated by condensing various carbonyl compounds with NH_2_OH·HCl under optimized reaction conditions using the ball milling technique. As shown in Table 2, the present method is quite general and practical for forming the aldoximes and ketoximes.

The lower yields within longer reaction times were observed for the aldehydes bearing electron-donating substituents than those containing electron-withdrawing substituents. Furthermore, 2-nitrobenzaldehyde and 2-methoxy benzaldehyde gave the highest and lowest yields (Table 2, entries 5 and 7). These results are assigned to the displacement of the aldehyde group from the plane of the aromatic ring by the ortho substituent, which, in turn, causes an increase of the electrophilicity of ortho-isomer than that of meta- and para-isomers [37]. Cinnamaldehyde was smoothly converted to the corresponding oxime without any by-products (Table 2, entry 11). However, it is worth mentioning that the ketoximes were obtained in a lower yields and a little longer reaction times (Table 2, entries 12–14).

PVIm was easily separated from the reaction mixture by simple filtering, washed with toluene, and dried at 80 °C under vacuum. Then, the model reaction was carried out using recovered PVIm several times, giving the desired oxime in 94, 94, 93, and 92% yields during four runs within 30 min (Table 2, entry 4). This result displayed the practical and high recyclability of PVIm in the current catalytic synthesis of oximes.

**Scheme 2:**
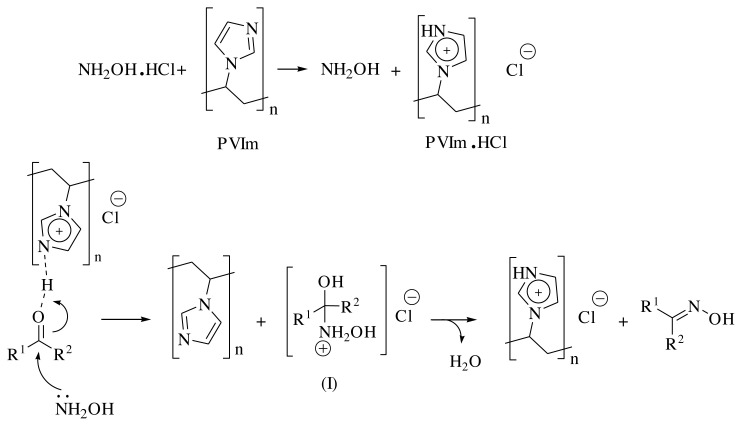
A schematic route for the synthesis of oximes in presence of PVIm under solvent-free conditions.

Based on a literature survey, a schematic route of the mechanism is illustrated for the current catalytic synthesis of the oximes. It is indicated that PVIm can act as a scavenger of HCl and liberate slowly and moderately an amount of hydroxylamine from its salt, which has a chance to react with the carbonyl compounds before leaving the reaction mixture. As shown in Scheme 2, PVIm acts as an HCl scavenger to liberate hydroxylamine and simultaneously activates the carbonyl group for the nucleophilic attack of the released hydroxylamine. Finally, PVIm can promote dehydration of intermediate **I**, which gave the corresponding oxime. The low temperature, solvent-free condition, and the formation of PVIm·HCl suppress the possible hydrolysis of the obtained oximes, which can be promoted in the presence of free hydrochloric acid and normal environmental humidity in the laboratory.

## 4. Conclusion

In summary, an efficient and greener catalytic process was developed to synthesize oximes using poly(4-vinylimidazole) as an easy separable and highly recyclable heterogeneous catalyst through the ball milling technique. The PVIm could be recovered simply by biphasic separation and reused several times without a considerable drop in the yield of the desired products.

## Figures and Tables

**Table 1:** The effect of PVIm loading and reaction time on the reaction of 4-nitrobenzaldehyde with hydroxylamine hydrochloride.^a^

Entry	Catalyst (mg)	Time (min)	Yield (%)^b^
1	-	60	-
2	100	30	67
3	100	60	78
4	150	30	74
5	150	60	86
6	250	30	94
7	250	60	96
8	500	40	95

a Reaction conditions: 4-Nitrobenzaldehyde (5.0 mmol), hydroxylamine hydrochloride (6.0 mmol), room temperature, solvent-free.

b Determined by GC-MS.

**Table 2:** The substrate scope of solvent-free condensation of various carbonyl compounds with NH_2_OH·HCl using PVIm by planetary ball mill technique.

Entry	Carbonyl compounds		Oximes	Time (min.)	Yield (%)^a^	M.p. (ºC)		Ref.
	R^1^	R^2^				Found	Reported	
1	C_6_H_5_-	H	2a	50	91	Oil	29–31	32
2	4-Cl-C_6_H_4_-	H	2b	50	94	58–59		32
3	4-CH_3_-C_6_H_4_-	H	2c	50	92	72–74	76–78	33
4	4-O_2_N-C_6_H_4_-	H	2d	30 (30, 30, 30)^c^	94 (94, 93, 92)^c^	132–134	125–127	32
5	2-O_2_N-C_6_H_4_-	H	2e	45	96	110–112	98–102	35
6	4-CH_3_O-C_6_H_4_-	H	2f	50	78	37–39	39–42	32
7	2-CH_3_O-C_6_H_4_-	H	2g	65	74	84–86	87–89	32
8	4-(CH_3_)_2_N-C_6_H_4_-	H	2h	120	86	135–137	139–141	32
9	Salcilaldehyde	H	2i	70	82	109–111	110–116	34
10	Terephthaladehyde	H	2j	90	78	201–203	202–204	34
11	Cinnamaldehyde	H	2k	45	84	137–139	135	35
12	Cyclohexanone		2l	120	77	86–88	91	36
13	C_6_H_5_-	CH_3_-	2m	90	79	52–54	55–60	36
14	C_6_H_5_-	C_6_H_5_-	2n	90	62	140–142	140–144	36

a Isolated yields.

b Reaction conditions: carbonyl group (5.0 mmol), hydroxylamine hydrochloride (6.0 mmol), PVIm (250 mg), room temperature, solvent-free.

c The yield and the reaction time using recovered PVIm.
